# Complex European invasion history of *Anoplophora glabripennis* (Motschulsky): new insights in its population genomic differentiation using genotype-by-sequencing

**DOI:** 10.1038/s41598-024-54567-y

**Published:** 2024-02-21

**Authors:** Iris Haeussermann, Martin Hasselmann

**Affiliations:** Institute of Animal Science, Department of Livestock Population Genomics, Centre for Biodiversity and Integrative Taxonomy (KomBioTa), University of Hohenheim, Stuttgart, Germany

**Keywords:** Genotyping and haplotyping, Population genetics

## Abstract

Anthropogenic activities like trade facilitate increasing rates of biological invasions. Asian long-horned beetle (ALB), which is naturally distributed in eastern Asia (China, Korean peninsula), was introduced via wood packing materials (WPM) used in trade to North America (1996) and Europe (2001). We used 7810 single nucleotide polymorphisms (SNPs) derived by a genotype-by-sequencing (GBS) approach to decipher the introduction patterns into Europe. This is applied for the first time on European ALB outbreaks from Germany, Switzerland, and Italy, both from still active and already eradicated infestations. The genome-wide SNPs detected signs of small and highly structured populations within Europe, showing clear founder effects. The very high population differentiation is presumably derived from multiple independent introductions to Europe, which are spatially restricted in mating. By admixture and phylogenetic analyses, some cases of secondary dispersal were observed. Furthermore, some populations suggest admixture, which might have been originated by either multiple introductions from different sources into the new sites or recurrent introductions from an admixed source population. Our results confirmed a complex invasion history of the ALB into Europe and the usability of GBS obtained SNPs in invasion science even without source populations.

## Introduction

Biological invasions increase worldwide, together with global trade of commodities and travel activities^1^. Compared to natural invasions, those which are facilitated by anthropogenic factors occur in much higher rates, with a higher chance of secondary dispersal^2,3^. Especially insects are very easily transported accidentally by humans due to their small size^4^. For instance, juvenile stages of cerambycids can infest wood-packing materials (WPM) such as pallets, crates, or dunnage. With little or no sign of infestation from outside of WPM, visual inspections alone are not enough to detect infested materials^5^. The Asian long-horned beetle (ALB), *Anoplophora glabripennis* (Motschulsky, 1853), is a xylophagous wood-boring beetle from the family of Cerambycidae with a wide range of host trees. Native to East Asia (China, Korean peninsula), ALB is a very destructive invasive species worldwide, primarily dispersed by WPM as vehicle. ALB was introduced and established outside its native range in North America and Europe, leading to extensive economic and ecological damage in every infestation site^6,7^.

Early responses to a biological invasion event are the most cost-effective approaches to minimize risk, and the inclusion of state-of-the-art genomic approaches improves the diagnostics enormously^8,9^. The identification of dispersal pathways can be difficult when considering the extent and heterogeneity of transported commodities^10^, but it is also challenging to uncover the complex evolutionary processes during biological invasions^11^. Anthropogenic driven invasions mostly have very complex introduction routes^12^. Hence, reconstructions of introduction routes promote a better understanding of the evolutionary processes^13^ and support reliable prediction models^14^. Previous studies using mtDNA markers (COI) and microsatellites to study the dispersal pattern of *A.* *glabripennis* into and within Europe have indicated the patterns are highly complex and probably involved multiple independent introductions and some cases of human mediated secondary dispersal^15,16^. Genetic structure in the native range from China and South Korea, as well as in the invasive range of North America could also be observed by such markers (COI, COII, microsatellites) but was not completely discriminated before^17–21^. Although all these studies facilitated insights in the population structure and dispersal patterns, they only provide relatively low-resolution information on single outbreaks and are limited in the precise reconstruction of introduction histories.

Consequently, thousands of genome wide SNP markers obtained by high-throughput sequencing (HTS) provide a high level of resolution and substantially improves the understanding of invasion process of invasive alien species (IAS)^8,9^. In particular, present and past population processes can be elucidated in more details as e.g. Rašić et al*.*^22^ provided remarkable insights into broad-scale population structure and fine-scale relatedness using ddRAD sequencing on *Aedes aegypti*.

For many cases, whole genome sequencing approaches are not necessary, thus HTS methods are often concomitant with a reduction of the complexity and genome representation like restriction-site-associated DNA sequencing (RAD-Seq) and Genotype-by-sequencing (GBS)^23,24^. Especially, methods like GBS make HTS even more feasible with the opportunities to genotype a vast number of individuals at thousands of SNP markers at the same time^24^. Thereby, thousands of variants are genotyped for samples across the genome, leading to SNP-markers suitable for comprehensive population genetic analyses^8^. With this method, population structure can be investigated without prior knowledge about the genome of the species of interest^24^. The GBS method was successfully applied to characterize the introduction patterns of the invasive gypsy moths *Lymantra dispar* *asiatica* and *L. d. japonica* to North America, and geographic variants, subspecies and introgression events were detected^25^. Recently, Cui et al*.*^26^ published data on population structure of native ALB populations using genome-wide SNP markers. This study aimed to disentangle the historical movements between regions in the native range to develop applicable biosurveillance tools. Using 6102 informative SNPs and 53 microsatellites, they determined six distinct population clusters among the native ALB populations and a clear separation between South Korean and Chinese populations. Hence, this method is promising for the investigation of introduction patterns *of A.* *glabripennis* in Europe, too.

**Figure 1:**
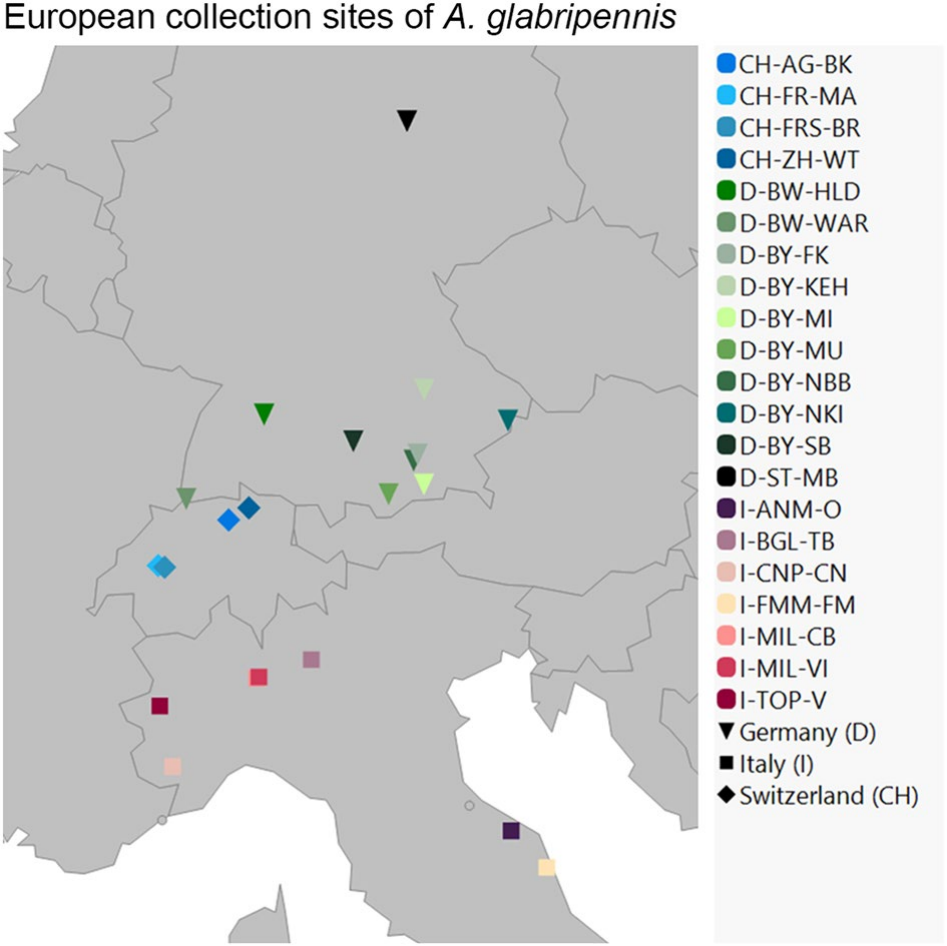
European collection sites of ALB used in this study. The codes of the populations (Pop-ID) are explained in the supplemental material (Table S5).

In this study, the normalized GBS (nGBS) protocol of LGC Genomics GmbH (Berlin, Germany) was used, which is applicable to any genome without the need of prior knowledge and is unaffected by genome size, methylation patterns or repetitive sequences. Commonly, for new species, several trials for the appropriate RE combination must be performed in advance, whereas nGBS uses for all genomes the blunt end cutter RE MsII^27^. This method was applied on 168 individuals (114 Germany, 20 Switzerland, 31 Italy, 3 China). The collection sites are depicted in Fig. 1 with further descriptions in Table S5 (Supplemental Material). To exclude batch effects of separate three GBS libraries, the specimen D-BY-SB-15-047 was sequenced in all runs as internal control.

## Results

### SNP genotyping

We obtained variant call files of three GBS libraries from LGC Genomics GmbH (Berlin, Germany) from 185 individuals (183 individuals and 2 internal controls) in total, and 331191 SNPs were identified after merging. The evaluation of the dataset on batch effects (non-biological factors) resulted in no or negligible effects of the library (Supplemental Material, Figs. S1–S3). This conclusion is mainly supported by a consistent pattern on population stratification (PCA) between minor and strict filtered datasets (Supplemental Material, Table S1), as well as by consistent results for the internal controls. Measures of mean individual depth and missingness initially indicated a separation by the different GBS libraries, which disappears by stricter filtering. After filtering (Supplemental Material, Table S1), we kept 7810 SNPs from 170 individuals (114 Germany, 20 Switzerland, 31 Italy, 3 China. 2 internal controls) for population genomic measures and phylogeny.

Figure S4 (Supplemental Material) pictures a moderate background and very slow the decay of the linkage disequilibrium (LD) with the increasing distance of the SNPs to each other, shown with distances from 0 to 99 kb with bins of 1 kb. The value 0.15 was used as a threshold to determine the independent SNPs for filtering by LD.

### High population structure of invasive ALB populations in Europe

**Figure 2:**
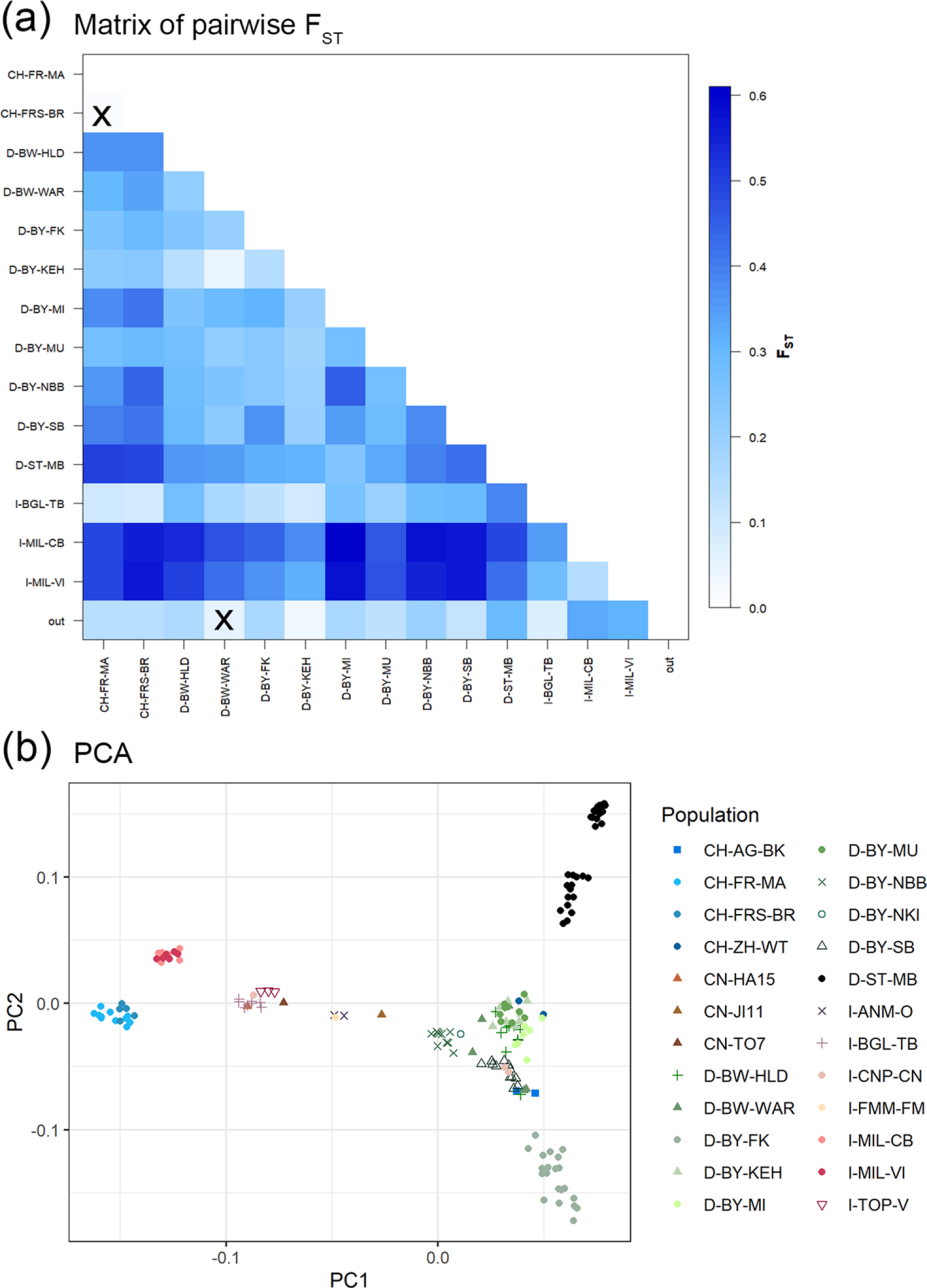
Measures of population structure of invasive ALB populations in Europe (**a**) Pairwise F_ST_-values among populations. x = not significant (significance level = 0.05) based on the LD-pruned subset of genomic ALB SNP data with 7810 SNPs. (**b**) Scatter plot of principal components PC1 (17.513%) and PC2 (11.002%) on the LD-pruned subset of genomic ALB SNP data with 7810 SNPs. Population IDs used here are described in Supplementary Table S5.

As a measure of intraspecific genetic differentiation, the pairwise F_ST_-values between the populations, as well as the Analysis of molecular variance (AMOVA) were conducted in Arlequin v.3.5^28^. For these estimations, only populations with at least 5 specimens were considered. The AMOVA tested no genetic structure and a country-wise genetic structure with the assumption of no differentiation within and between groups. The global AMOVA results are demonstrated in Table 1 as a weighted average over 4331 loci. This method revealed very high overall F_ST_-values of ~ 0.35–0.44 between the populations in the tested scenarios. The non-hierarchical analyses estimated ~ 35% of the variation between the populations and ~ 65% of the variation within the populations. Grouping according to countries revealed the lowest molecular variation between the countries (~ 16%), whereas the highest genetic diversity was within the populations (~ 56%) and with ~ 27% slightly lower between the populations within a country. The fixation indices F_ST_, F_SC_ and F_CT_ are also shown in the description of Table 1. The highest degree of differentiation was estimated in both tested scenarios with F_ST_-values to show the differentiation among the populations with ~ 0.35–0.44. The differentiation between the populations within a country (F_SC_) was very high with ~ 0.33, whereas the degree of differentiation among the countries (F_CT_) was relatively moderate (~ 0.16). The pairwise differentiation between the sampled populations was measured in pairwise F_ST_-values based on genotype frequencies. All specimens from populations with less than 5 individuals were assigned to the group "out". The pairwise F_ST_ matrix is pictured in Fig. 2a with grades of coloring from low to high, while the exact F_ST_-values and the corresponding p-values are shown in the Supplemental Material (Tables S2–S3). For most of the pairwise F_ST_-values between the sampled populations, high (0.15–0.25) to very high genetic differentiations over all sites (> 0.25) were estimated. Moderate genetic differentiation (~ 0.05–0.15) was examined between Kelheim (D-BY-KEH) and Trescore-Balneario (I-BGL-TB), Kelheim and Weil am Rhein (D-BW-WAR), Trescore-Balneario and Marly (CH-FR-MA)/Bruensried (CH-FRS-BR), as well as between Marly and Bruensried (not significant). Trescore-Balneario showed considerably moderate differentiation to Weil am Rhein with F_ST_-values slightly above 0.15 but < 0.2. Very strong genetic differentiation to all the other populations was detected in Marly and Bruensried. Corbetta (I-MIL-CB) and Vittuone (I-MIL-VI) were also strongly differentiated to the other populations with F_ST_-values > 0.4 and even some with > 0.5.

**Table 1:** Global AMOVA results as a weighted average over 4331 loci (LD-pruned ALB SNP subset with 7810 SNPs).

Source of variation	Sum of squares	Variance components	Percentage variation
(a) AMOVA—no. of groups 1			
Among populations	72,168.004	291.602 (Va)	34.684***
Within populations	128,889.709	549.148 (Vb)	65.316
Total	201,057.714	840.750	
(b) AMOVA—no. of groups 3—CH, D, I			
Among groups	21,676.457	151.379 (Va)	16.394***
Among populations within groups	47,618.770	253.354 (Vb)	27.438***
Within populations	107,718.758	518.636 (Vc)	56.168***
Total	177,013.985	923.369	

(a) no hierarchical structure (b) structure to test: populations of Switzerland, Germany and Italy grouped to the respective country.

Significance tests (1023 permutations) for all Va, Vb, Vc and fixation indices: ***p ≤ 0.001; (a) F_ST_ = 0.347***, (b) F_ST_ = 0.438***, F_SC_ = 0.328***, F_CT_ = 0.164***.

A principal component analysis (PCA) was performed on the LD-pruned dataset with 7810 independent SNPs. The first two PCs together explain 28.5% of the variation of the data, since PC1 represents 17.5% and PC2 11.0% of the variation (Fig. 2b). PC3 (9.3%), PC4 (6.9%) and PC5 (6.5%), which combined explain 22.7% of the variation can be found in the Supplemental Material (Figs. S5–S7), as well as the Eigenvalues (Table S4, Fig. S4). The Swiss and Italian clusters (CH and I) on the left are separated from all German (D) and some Swiss collection sites. One of the separated groups contains all specimens from the Swiss locations Marly (CH-FR-MA) and Bruensried (CH-FRS-BR), another one all specimens from Corbetta (I-MIL-CB) and Vittuone (I-MIL-VI), Italy. Another clearly separated group contains Italian specimens from Vaie (I-TOP-V), Trescore-Balneario (I-BGL-TB), Cuneo (I-CNP-CN) and the two Chinese specimens from Harbin and Tongliao (CN-HA, CN-TO). The two specimens from Ostra (I-ANM-O) were separated with the single specimen from Fermo (I-FMM-FM), while Jinan (CN-JI) stands for itself nearby. In the upper right part of the plot, two clusters from Magdeburg (D-ST-MB) stand out. On the bottom right side of the plot, Feldkirchen (D-BY-FK) forms a cluster with all its specimens. In the coordinates aggregated around zero-point, all other populations were represented. In Fig. S5, it is observable that PC3 separated all specimens from Schoenebach (D-BY-SB; including the internal controls).

### Ancestry of European invasive ALB populations

**Figure 3:**
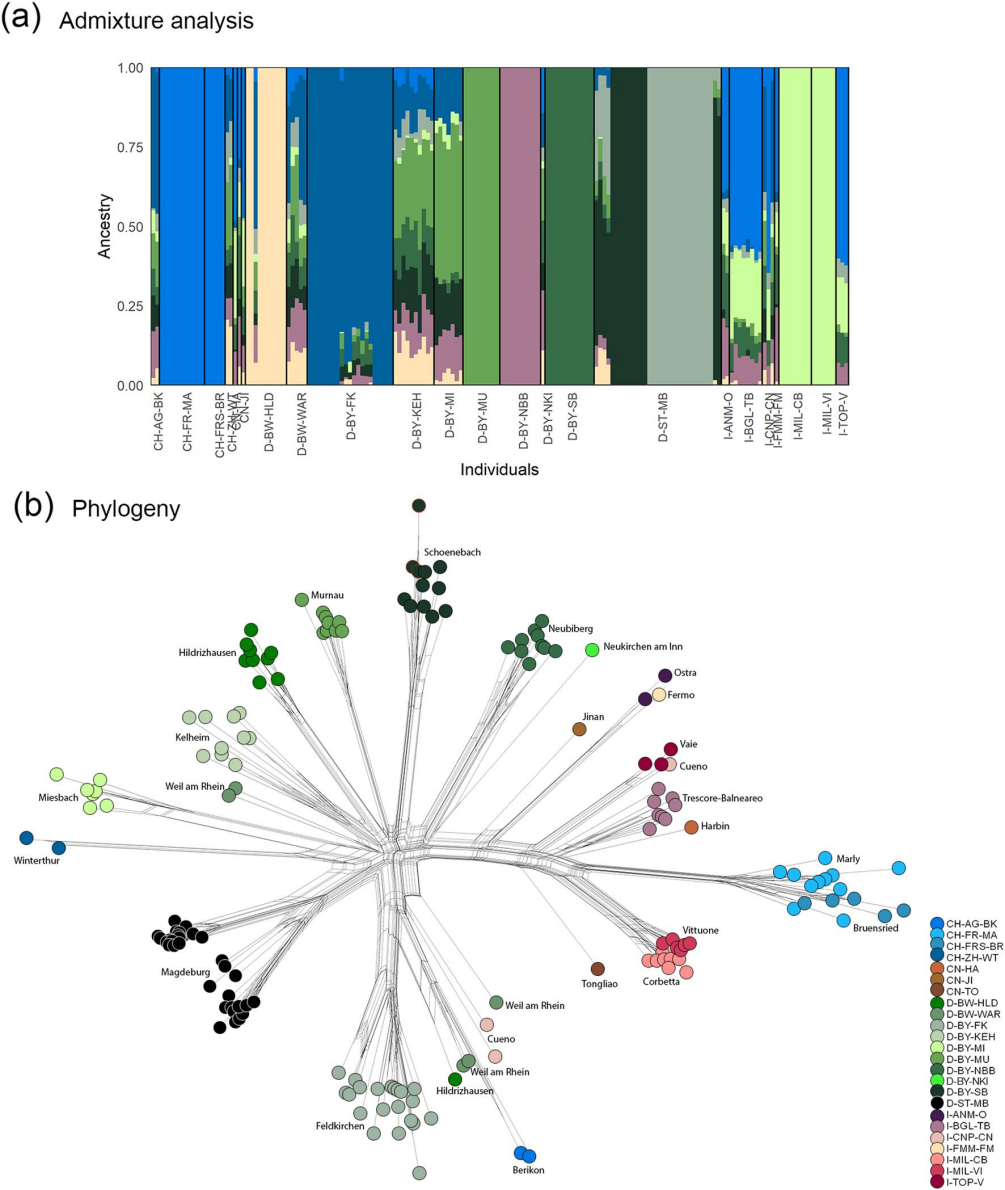
Analyses of individual and overall kinship (**a**) Bar plot of analysis of ancestry of 170 ALB samples from Switzerland, China, Germany, and Italy conducted in ADMIXTURE v.1.3.0; K = 9; 7810 SNPs. Vertical bars represent each specimen, while colored segments represent the proportion of ancestry to the different clusters. (**b**) Phylogenetic network using ɣ-distributed (+ G 2.69) K2P distances with invariable sites (+ I 0.05) and Equal Angle; Fit = 95.925; 170 taxa and 7810 SNPs are shown. The three red framed specimens represent the internal controls from three GBS runs.

To determine the individual kinship, an admixture analysis was performed with a dataset of 7810 independent SNPs. While conducting the admixture analyses using the clustering software ADMIXTURE v.1.3.0, the cross-validation procedure was used to identify the optimal K (Supplemental Material, Fig. S8). The results of nine alleged populations (K = 9) are depicted in Fig. 3a. The results K = 2–11 from the same dataset are given in Supplemental Material, Fig. S9. It is clearly observable, that most of the individuals within a collection site are directly clustered together, which also is true for sites with proximity (Marly/Bruensried, Corbetta/Vittuone). For Murnau (D-BY-MU) and Miesbach (D-BY-MI) this was the case as well with K = 9, even though they are geographically about 60 km apart from each other. With K = 7 and K = 8, the populations Miesbach and Murnau showed different clustering patterns, as well as with K = 10 and K = 11. Interestingly, the specimens from Magdeburg (D-ST-MB) clustered in two clearly separated populations and one mixed population from specimens collected in 2015. Other locations showed individuals with an admixed ancestry represented by different contributions from populations, too e.g. Berikon (CH-AG-BK), Winterthur (CH-ZH-WT), all three Chinese specimens, one single specimen from Hildrizhausen (D-BW-HLD), Weil am Rhein (D-BW-WAR), Kelheim (D-BY-KEH), Neukirchen am Inn (D-BY-NKI), Magdeburg 2015 (D-ST-MB) and several Italian specimens. Smaller portions of another population represented in addition to a main one, were found in Feldkirchen (D-BY-FK), Miesbach (D-BY-MI) and in some Magdeburg-2019 specimens (D-ST-MB). The mixed populations from Switzerland and Germany consist of a similar genetic composition except the mixed ones from Magdeburg consisting of a unique composition. Mixed populations from Italy are composed similarly, too. Each of these mixed populations contain larger parts, which are also found in Marly and Bruensried in Switzerland.

The NeighborNet phylogenetic network (Fig. 3b) drawn with Splitstree v. 4.17.1 provides additional insights of evolutionary relationships. For instance, the separation in three groups of all samples from Magdeburg (D-ST-MB) that cluster to the same origin. Similar separation can be observed for Feldkirchen (D-BY-FK) in two different groups, which both share crosslinks at the same origin at a longer distance together with single specimens from Hildrizhausen (D-BW-HLD), Weil am Rhein (D-BW-WAR), Berikon (CH-AG-BK) and Cuneo (I-CNP-CN). Especially, some single specimens from one collection site strikingly occur at multiple different positions in the phylogenetic network like from Hildrizhausen (D-BW-HLD), Weil am Rhein (D-BW-WAR) and Cuneo (I-CNP-CN). The very clear separation of most of the Italian findings together with the findings of Swiss Marly (CH-FR-MA) and Bruensried (CH-FRS-BR) are also standing out here, supporting the PCA results. They all share cross-links in the directions of the root together with the three Chinese specimens, but all of them show a clear separation at certain points. The three specimens from China are all standing alone in this network but are in general genetically closer to populations found in Italy, and Harbin occurred closest to the findings from Trescore-Balneario (I-BGL-TB). For the neighboring sites Marly/Bruensried and Corbetta/Vittuone which showed the same ancestry in the admixture plots, a small separation became apparent in the network. Also, the two specimens from Ostra (I-ANM-O) and Fermo (I-FMM-FM) share the same branch. One specimen from Hildrizhausen and two from Weil am Rhein have a different branch but share cross-links at the beginning. All other specimens clustered together with the other specimens from their own collection site with tight cross-links. An exception to this are the specimens from Kelheim (D-BY-KEH) where each specimen has an own branch respectively that roots together near to the midpoint. Similar observation was made for Trescore-Balneario, but with less genetic divergence compared to Kelheim. In general, all the populations from Germany, Winterthur (CH-ZH-WT), Berikon (CH-AG-BK) and two specimens from Cuneo root in the middle in a similar branch length to each other. Thus, Marly and Bruensried are the most genetic divergent sites compared to the root of the German and other Swiss populations.

## Discussion

We used nGBS for population genomic insights of *A.* *glabripennis* revealing kinship and population structure between different infestation sites to infer introduction patterns into Germany, Switzerland, and Italy. The dispersal pattern of *A.* *glabripennis* into and within Europe is proven to be shaped by multiple independent introductions and some human mediated secondary dispersal, as also indicated before^15,16^. The presumably mostly single introduction events into Europe are concomitant with the evidence for population structure, resulting in a severe reduction of genetic diversity (data not shown). The European ALB SNP dataset showed a moderate LD with very slow decay of LD (Fig. S4, Supplemental Material) indicating small and bottlenecked founder populations within Europe. There was also a conspicuously high amount of very rare SNPs, which is likewise typical for a small random sample introduced by source populations. This phenomenon is called founder effect, when the frequencies of originally rare alleles is increased by genetic drift^29^. The results of the admixture analysis confirm this observation, displaying almost all infestation sites with a homogeneous genetic structure within the site.

The extremely strong differentiation among all populations (~ 0.35–0.44 F_ST_) suggests no mating and mixture between most of the infestation sites within Europe. Furthermore, the differentiation and variation among the populations within a country (~ 0.33 F_SC_) suggest that introduction sources within the countries are likely not shared in most cases, hence the levels of secondary spread are indicated to be low. On the other hand, the high but comparatively lower differentiation among the countries indicates, that within Europe a huge spectrum of genetic backgrounds from the source populations is represented, from which some came from shared introduction sources or introductions with less differentiated source populations. The pairwise F_ST_-values between the European populations validated the measured population structure in more detail. Moderate differentiation was only measured between the neighboring locations Marly-Bruensried and between populations that showed high proportion of populations in the admixture analysis (Fig. 3a). This is either a sign of a human mediated secondary dispersal, the same source of introduction, or some other shared proportion of genetic ancestry. Surprisingly, Corbetta and Vittuone showed some population structure (F_ST_ = 0.149). Hence, these populations have variance in the SNPs, as also visible in the small separation of the two populations in the phylogeny (Fig. 3b). According to the proximity, the variance is probably stemming more from genetic drift in the bottlenecked founder populations than from genetic structure.

The admixture analysis models the proportions of the genome derived from different source populations^30^ and the ancestry (Fig. 3a) revealed for most individuals the same population(s) within their collection site, which is a sign of one introduction and genetic drift. Hence, even the collection sites with several population proportions were in the most cases homogeneous within the sites. The phylogenetic network analysis confirmed this observation. Beside a few exceptions, most specimens were clustered together by collection site and are tightly cross-linked, reflecting the strong kinship within the collection sites, most likely due to a small amount of founding individuals, as observed before in Europe^15^. Evidence of secondary translocation and high population structure within the European infestation sites of ALB could also be confirmed. Apparently, most of the individuals within a collection site were directly genetically relatable and differentiated from other infestation sites. This is most likely the result of non-random mating within Europe and can also be a sign of severe genetic bottlenecks. In populations with only one dominating population proportion, this could have also been the only introduced subset from a source population and got fixed in the new invasive population by genetic drift and/or preferred mating of some individuals^29,31–33^. The branching pattern in phylogeny of the specimens found in Weil am Rhein, Cuneo and Hildrizhausen assume either multiple introductions from different sources, or recurrent introductions of a genetically diverse source.

In the case of the neighboring sites Marly and Bruensried, as well as Corbetta and Vittuone, it can be assumed that there was secondary transfer between those sites, as it was already reported by Tsykun et al*.*^16^ from Marly to Bruensried and by Javal et al.^15^ for Furiani in Corsica. Also, Fermo and Ostra, which are 90 km apart, indicate one introduction and secondary dispersal or the same source. Possibly, this could also be the case between Murnau (D-BY-MU) and Miesbach (D-BY-MI), which are 60 km apart. On closer inspection of several potential population compositions, a secondary dispersal seems comparatively unlikely between Murnau and Miesbach, since the population proportions with K = 7–8 and K = 10–11 differ, whereas in the cases of Marly-Bruensried and Corbetta-Vittuone the clustering was stable in different K (Fig. S9, Supplemental Material). The serial increased K on the admixture analysis (Fig. S9) underline the topologies drawn by the PCA (Fig. 2b) and phylogeny (Fig. 3b) showing an ancestral split from most Italian and Swiss specimens from the German, few Swiss and few Italian specimens. This observation suggests presumably two different geographic larger source regions within the native range, which were not admixed with each other. According to the recent study of Cui et al*.*^26^ this might reflect the clear distinct northern and southern groups, or the clear separation of most populations from South Korea and China. Each of the two groups are highly differentiated between the collection sites but have a shared proportion of genetic ancestry. Assuming South Korea as source for the Marly/Bruensried infestation as suggested by Javal et al.^15^, it can be speculated that most Italian and Swiss specimens from this study originate from the area of North eastern China (to the east of gulf of Bohai) and South Korea, while the rest originated from Western and central China. An analysis of the specimens from this study with the SNP panel obtained by Cui et al.^26^ could solve this open question.

The admixture analysis showed mixed populations, which differ between Italy/China and Germany/Switzerland in their population composition. Potential explanations are recurrent introductions from a strongly admixed source, or different sources and subsequent admixture in Europe. For Cuneo and Weil am Rhein, multiple introductions from different sources are probable, since single specimens are differently placed in the phylogeny. Otherwise, particularly the fan-shaped structure in the phylogeny of Kelheim and Trescore-Balneario suggests either multiple introductions and subsequent mixture in Europe or consecutive introductions keeping the propagule pressure high and maintaining genetic diversity of one introduction source. Additionally, there is also the possibility of a dataset bias resulting from a related unsampled population which is closely related to several other populations. Hence, the ancestry might be incorrect since the algorithm always fits the data into a pattern of admixture proportions^34^, which would not be possible to be proven due to the shortage of specimens from native populations. The cases of Feldkirchen/Magdeburg, where the phylogeny revealed a separation into two (FK)/three (MB) groups, could also result from one admixed population from which specimens dispersed in different city parts and were thereby bottlenecked. Alternatively, two (FK)/three (MB) introduction sources that are regional in close vicinity could still have emerged from one introduction from regional export hubs. Several introductions from different regions would be least parsimonious. The more likely first scenario is only possible with several generations prior to the first detections (FK 2012; MB 2014), which is not unusual for IAS^35^.

Given the complex ALB invasion history into Europe, the lack of representative samples from native/invasive source populations limits the deviation of introduction routes. The three sampled Chinese locations Harbin (CN-HA), Jinan (CN-JI) and Tongliao (CN-TO) do not seem related to the studied European populations, limiting further conclusions to be drawn. Maybe Harbin is in proximity of the introduction source of Trescore-Balneario, if the case of consecutive introductions of a diverse introduction source is true, which cannot be deciphered with this dataset. In most cases the introductions into Europe were presumably independent, strictly separated, and due to the strong detected bottlenecks and founder effects by genetic drift not connected with any other ALB population in Europe. The phylogeny network contains several basal connections, which can be either ancestral splits or connections of former distinct groups due to the admixture in the native range. The founder effect could have been diminished by recurrent introductions with higher propagule pressure^29,36^, which might be the case in populations like e.g. Magdeburg or Feldkirchen.

For several collection sites, only a limited number of individuals could be obtained, limiting populations genetic conclusions. Irrespectively, for all 168 specimens we obtained individual kinship and phylogenetic information. To obtain robust insights from introduction sources, an appropriate sampling size, including natural origins, is mandatory^8^.

For this study, several infestation sites within Europe were sampled for population genetic analyses and have been analyzed by genome wide SNP markers for the first time. We obtained new insights into the invasion history within Germany and Italy and extended the results from Tsykun et al*.*^16^ in Switzerland. The resolution could be enhanced compared to previous studies and offers a valuable and highly reliable pattern of genetic relationships between German, Italian and Swiss infestation sites, but astonishingly, the inference of the European SNP dataset came to comparable conclusions as former studies using COI and microsatellites before^15–19,37^. All the previous studies emphasized the complexity of the invasive pathways of *A. glabripennis*, which are shaped by a highly indistinct genetic background from the native range and multiple independent, maybe consecutive introductions combined with secondary spread events in the invasive range. Most likely, these conditions, influenced by human-mediated translocations, fostered the invasion success of ALB in North America and Europe, since the limited genetic diversity did not attenuate the invasiveness^15,16,19^. Some of the mixed populations in the European dataset from this study are in accordance with the strongly admixed genetic background from Chinese ALBs as suggested by the previous studies. Carter et al.^21^ and Javal et al.^15^ proposed the genetic background from China to be shaped by complex migrations of *A.* *glabripennis* caused by humans and mixture from different sources during the reforestation program of the Chinese government, starting in the 1960s^6,38^. Cui et al.^26^ suggest that recent admixture in the native range occurred less than previously expected since they described contemporary movement between some regions in the native range but found no evidence for large-scale admixture. However, in our study probable admixture in the invasive range was reported for the first time (e.g. Kelheim, Trescore-Balneario), which either happened in the source populations prior to the invasion (e.g. in China), or within the respective European infestation site, or is at risk to happen there (e.g. Cuneo) because of multiple introduction sources. ALB is mostly dispersed in large spatial scales across countries and continents by human transport (containers, wood packing material, transport of wooden clippings, etc.), rather than among locations of one invaded country. However, exceptions like Marly/Bruensried and Corbetta/Vittuone argue for either human-mediated or natural range expansion within a small spatial scale, which was also described in previous studies^15,16,18^.

Despite the evidence of scarce genetic diversity within most infestation sites due to population bottlenecks, *A.* *glabripennis* could establish in many infestation sites, at least for a while in case of already eradicated sites. The sites with recurrent or multiple introductions have most likely not much loss of genetic diversity. Anthropogenically induced adaptation to invade is a very likely scenario, for which reason ALB was not much affected by hostile effects from genetic bottlenecks^39^. The natural, not invasive populations in China/South Korea from rural or forest areas are improbable introduction sources because of reduced connectibility. Transportation hubs, which accelerate spread^40^, are in proximate distance to (sub)urban infestation sites in China/South Korea that are most likely themselves invasive^18,20,37^. In Europe, ALB does not face adaptive challenges because the climate conditions and host trees are comparable to introduction sources from (sub)urban areas in Asia, and it is known that matching environments can promote invasion success (e.g.^41^). Admixture events could have created new genetic variation and therefore may have strengthened the populations^20,42,43^.

The multitude of different quality and storage levels of the received ALB samples from the federal plant health offices disclose the necessity of a reformation for advanced future practical approaches. Even though keeping specimens pinned is important for training and educational purpose, it should not be the preferred practice for sampling as subsequent, mandatory genetic analyses are hampered. Storing of specimens at − 20 to − 80 °C and with appropriate sample amounts per collection site (at least 10) that are solely dedicated for DNA-based approaches, along with keeping morphological references, should be the norm. For genome sequencing approaches, immediate shock freezing at the collection site would be most suitable. As proposed by Roe et al*.*^8^ and Blackburn et al*.*^44^, international frameworks and cooperation engaging NPPOs for sample collection and research institutions (e.g., Universities) for respective analyses are very promising to counteract the global spread of IAS. To merge all collected knowledge about biological invasions between different research institutions around the world, methods, standards, and data quality need to be aligned^44,45^ that include effective protocols for plant health practice to high quality genomic DNA. Further advice for phytosanitary measures based on the results of this study are special precautions when genetic analyses reveal multiple introduction sources, because these populations might be more invasive^29,36,46^. Hence, the strict restrictions in transport of wooden products in infested areas to avoid translocations in adjacent areas that already exist should be implemented very rigorously. Given that ALB usually flies only short distances, along with high distances among most collection sites, the results of this study are quite encouraging for the regional plant health offices, since they allow suggestions of no or only few human-mediated secondary translocations on medium or long distances.

## Methods

### Sampling of Asian long-horned beetle (ALB)

Asian long-horned beetle (ALB) samples were acquired from invasive European sites in Germany, Italy, and Switzerland, except three samples used for comparisons from the native range in China (received from Cui et al*.*^26^). Samples were collected from infestation sites in different years (2011–2019) and from members of federal plant health offices or research institutes. Some were received from still active infestations, whereas most sites are considered as eradicated by now. The samples consist of different developmental stages (larva, pupa, or imago) and different conditions of conservation (dried, fixed in ethanol and/or frozen in – 20 °C). Some samples from Magdeburg (Germany), the samples from Switzerland (obtained by Tsykun et al*.*^16^) and China were directly obtained as DNA extracts. Further details on the Pop-IDs and positions for each of the sampling sites are compiled in the supplementary material (Table S5) and the locations are shown in Fig. 1. We use the term population as samples belonging to one collection site.

### Genomic DNA preparation from ALB tissue

The CTAB protocol according to the description in Rusterholz et al*.*^47^ was identified to be most suitable for the different qualities and storing conditions of the tissue samples and amount of total DNA, with slight modifications. A precooled metal rack (– 80 °C) was used to freeze down the tissue sections (approx. 5–10 mg) for mechanical cell disruption using a micro pestle. Dried samples were incubated overnight instead of 90 min in the extraction buffer. For larval tissue, the extraction of the suspension with 500 µl chloroform/isoamyl alcohol (25:1) by inversions was repeated. In general, for adult beetles one leg was used for DNA extraction, if available. Otherwise, tissue parts of the thorax were used. From larvae, a small tissue section was used, avoiding the inclusion of intestine parts. Qubit® dsDNA BR Assay Kit (Thermo Fisher Scientific Inc., Waltham MA, USA) was used to determine the quantity of the genomic DNA extracts from beetle tissue in ng/µl with a Qubit Fluorometer. The quality was assessed by measuring the 260/280 and 260/230 ratios using a Nanodrop 2000/2000c Spectrophotometer. Molecular level of genomic DNA was determined by visual check after agarose gel electrophoresis, where some dried samples showed a higher proportion of low molecular, degraded DNA beside the high molecular DNA. Nevertheless, these samples were all used for sequencing as they passed minimum quality control and to reflect the reality of samples occurring in plant health controls.

### Genotyping and analysis of genome-wide SNP markers

Genotype-by-sequencing and SNP calling was conducted by LGC Genomics GmbH (Berlin, Germany) in three different runs using their specific normalized GBS protocol (nGBS)^27^. The DNA extracts sent to LGC Genomics GmbH were digested with the RE MslI before library preparation (insert size mean range: ~ 180–215 bp) and were run on a llumina NextSeq 500 for the paired-end sequencing (two times 150 bp).

To obtain genomic SNP markers via Genotype-by-sequencing approach, the reads were pre-processed and subsequently aligned with the reference genome of ALB (*A.* *glabripennis*: NCBI Agla 2.0^48^, https://www.ncbi.nlm.nih.gov/assembly/GCF_000390285.2) for calling the variants (SNPs). These steps were conducted by LGC Genomics GmbH (Berlin, Germany). The VCF-files received from LGC Genomics GmbH (Berlin, Germany) from three different runs of nGBS, were merged using VCFtools v. 0.1.15^49^. To evaluate the dataset on batch effects, measures for individual depth, missingness generated with VCFtools v.0.1.15 and principal components received from PLINK v.1.9^50,51^ were plotted for the raw data of the merged three GBS runs and filtered subsets. According to these results, raw VCF dataset was filtered based on phred scores, the missingness per individual and site as described in the Supplementals, Table S1.

Calculation of missingness per individual showed that most of the individuals had high relative count of missingness (F_miss value) between 0.6 and 0.7. Consequently, seven individuals with higher F_miss values than 0.9 were removed from the dataset. As the missingness per site in this dataset was very high too, the usual cut-off of 0.25 would not be proportionate for the available data. Therefore, the datasets were filtered on basis of the proportion of missing data to exclude sites with > 50% missingness.

To explore the measures of linkage, r^2^ was calculated in PLINK v.1.9^50^ using a window approach (-ld-window 100 -ld-window-kb 100 -ld-window-r2 0). The threshold for linkage disequilibrium (LD) with an r^2^-value of 0.15 was used, assuming all SNPs with an r^2^-value below 0.15 are in linkage equilibrium (LE) and independent. To prune the SNPs exceeding the set r^2^-threshold in PLINK v. 1.9 was used with a sliding window approach (25 kb windows and 5 kb step size).

### Population structure

The genetic structure within the dataset (7810 SNPs) was calculated, by means of the fixation indices F_ST_ and the molecular variance using Arlequin v.3.5^28^. For the AMOVA, the standard setting for AMOVA was used with 1000 permutations. Pairwise F_ST_ between populations was calculated with 100 permutations and a significance level of 0.05. Non-hierarchical AMOVA was used for testing a panmictic scenario and the hierarchical AMOVA was grouped by countries, excluding China. As another control measure of population stratification, a principal component analysis was performed in PLINK v.1.9.

### Analyses of individual and kinship

As a method of inferring the individual genetic ancestry to deviate the kinship of the analyzed specimens to each other, an admixture analysis using ADMIXTURE v.1.3.0^30^ was conducted on 7810 SNPs. Sequential admixture analysis was performed for K2-22, with additional cross validation. The phylogenetic network with Splitstree4^52^ was calculated using NeighborNet and EqualAngle. The distance method was γ-distributed Kimura-2-parameter with invariant sites (K2 + G + I) with α parameter for γ distribution 2.69, as well as proportion of invariable sites 0.05.

### Supplementary Information

Supplementary Information.

## Data Availability

The variant data for this study has been deposited in the European Variation Archive (EVA) at EMBL-EBI under accession number PRJEB66443 (https://www.ebi.ac.uk/eva/?eva-study=PRJEB66443).
