# Supplementary material for: Complex European invasion history of Anoplophora glabripennis (Motschulsky): new insights in its population genomic differentiation using genotype-by-sequencing
Source: Sci Rep. 2024 Feb 21;14:4263. doi: 10.1038/s41598-024-54567-y (PMC10881967; doi:10.1038/s41598-024-54567-y)
Supplement: Supplementary file 1 — Supplementary Information. [file 41598_2024_54567_MOESM1_ESM.docx]

# Results

## SNP genotyping


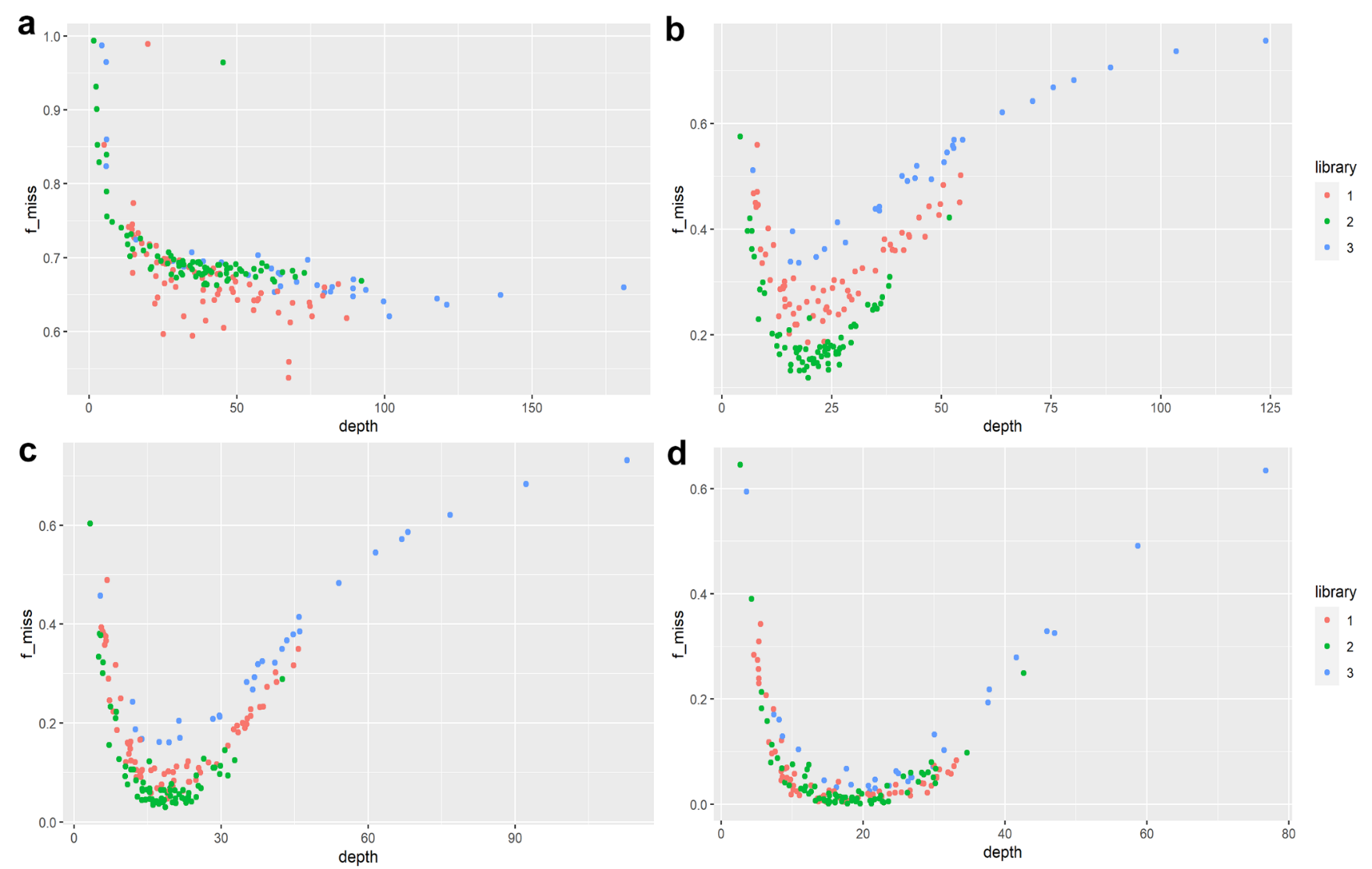


| **Figure S1.** Mean depth per individual (depth) plotted against the individual missingness (f_miss) coloured by library from the raw ALB SNP dataset with 185 individuals (a) and the different filtered data subsets b) 170Indvf8g50m50 c) 170Indvf8g75m25 and d) 170Indvf8g90m10. |
| --- |


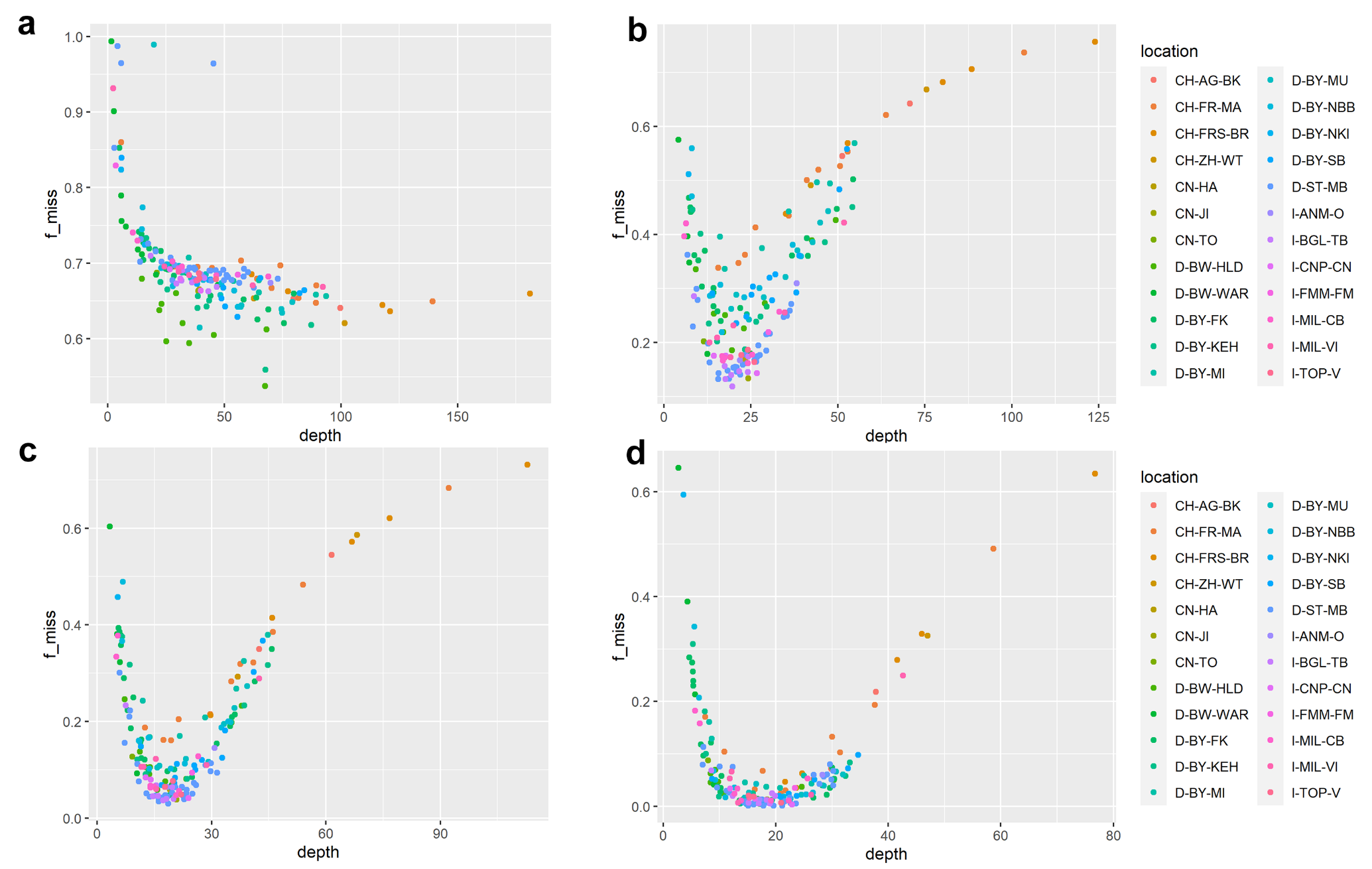


| **Figure S2.** Mean depth per individual (depth) plotted against the individual missingness (f_miss) coloured by location (Pop-ID) from the ALB SNP datasets: raw 185 individuals (a) and the different filtered data subsets b) 170Indvf8g50m50 c) 170Indvf8g75m25 and d) 170Indvf8g90m10. |
| --- |


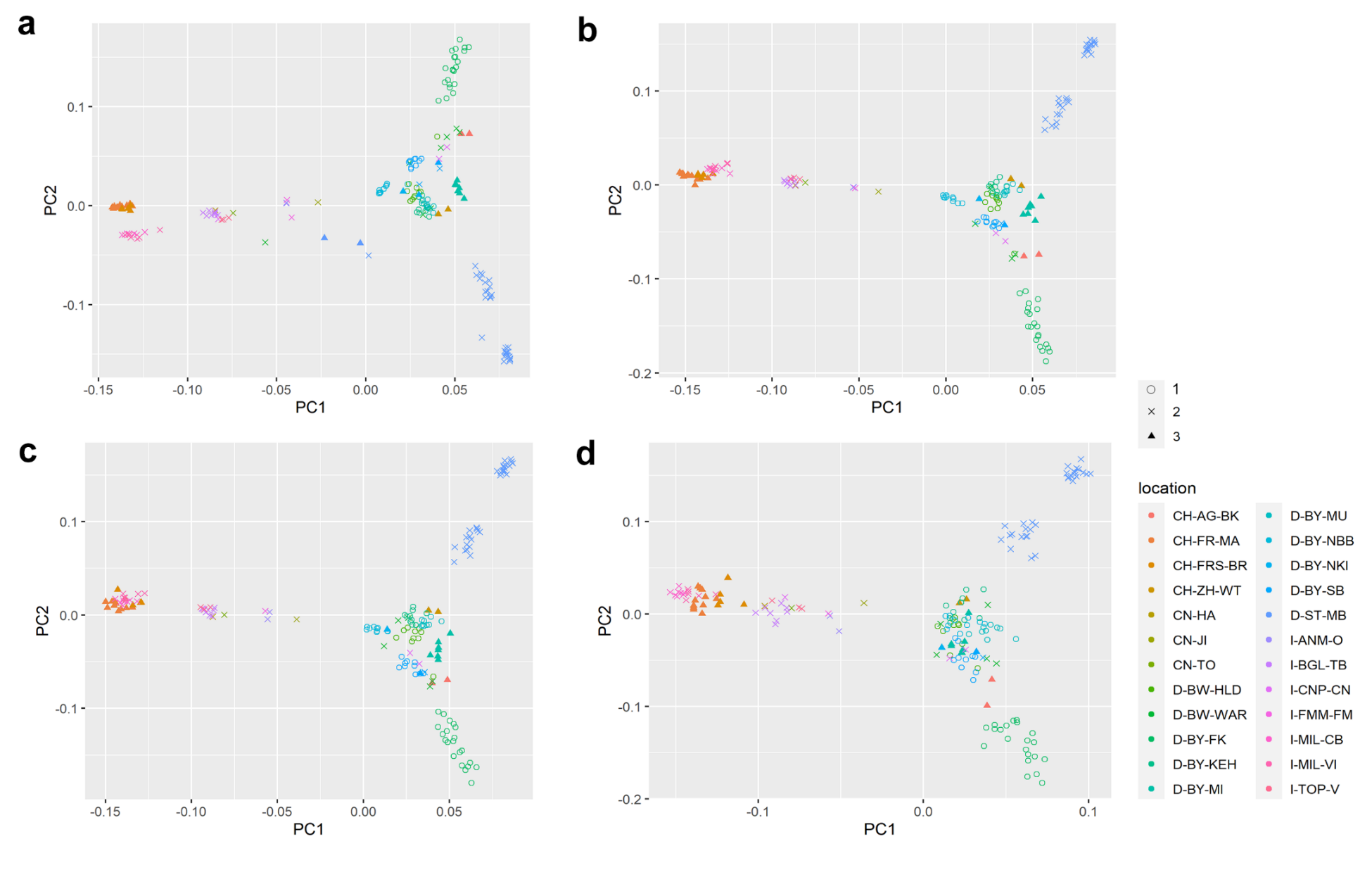


| **Figure S3.** Scatter plot of principal components 1 and 2 coloured by location and shaped by library from the raw ALB SNP dataset with 185 individuals (a) and the different filtered data subsets b) 170Indvf8g50m50 c) 170Indvf8g75m25 and d) 170Indvf8g90m10. |
| --- |

**Table S1.** SNP and individual count at all filter settings.

| VCF-file (.vcf) | SNPs | individuals | setting |
| --- | --- | --- | --- |
| GBS-Run1_Variants_raw_All.vcf | 239604 out of 239604 sites | 72 out of 72 | raw data Run1 |
| GBS-Run2_Variants_raw_All.vcf | 128391 out of 128391 sites | 80 out of 80 | raw data Run2 |
| GBS-Run3_Variants_raw_All.vcf | 180962 out of 180962 sites | 33 out of 33 | raw data Run3 |
| Variants_raw_185Indv.vcf | 331191 out of 331191 sites | 185 out of 185 | raw data merged |
| 178Indvf7.recode.vcf | 73364 out of 331191 sites | 178 out of 185 | filter7 |
| 170Indvf8.recode.vcf | 64830 out of 64830 sites | 170 out of 178 | filter8, Fmiss cut-off 0.7 |
| 170Indvf8g50m50.recode.vcf | 32432 out of 64830 sites | 170 out of 170 | max-missing 0.5 |
| 170Indvf8g75m25.recode.vcf | 10226 out of 64830 sites | 170 out of 170 | max-missing 0.75 |
| 170Indvf8g90m10.recode.vcf | 922 out of 64830 sites | 170 out of 170 | max-missing 0.9 |
| 170Indvf8g99m1.recode.vcf | 0 out of 64830 sites | 170 out of 170 | max-missing 0.99 |
|  |  |  |  |

filter7 (f7) = --minQ 30 --remove-filtered-all --maf 0.01 --mac 3 --max-alleles 2 --min-alleles 2 --min-meanDP 3 --minDP 3 --max-meanDP 55 --min-alleles 2, cut-off 0.9 on F_miss for individuals (--remove <file>); filter8 (f8) = --thin10 on f7-filtered subset; 185Indv = specimen count on the unfiltered merged VCF-file; 178Indv = specimen count after cut-off 0.9 on individual F_miss; 170Indv = specimen count after cut-off 0.7 individual F_miss on f8-filtered subset; g50m50 = genotype call rate 50% and tolerated missingness 50% (analogous for higher genotype rates); All individual counts include D-BY-SB-15-047 as internal control, for GBS-Run2 and GBS-Run3 only once, but in all following VCF-files three times respectively.


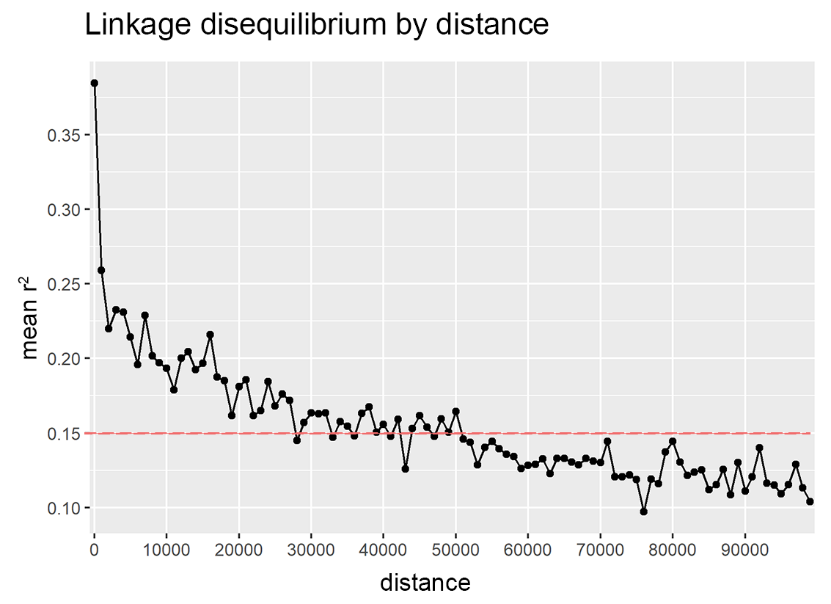


| **Figure S4.** Decay of linkage disequilibrium (LD) with physical distance (0-9.9 kb) on genomic ALB SNP subset 170Indvf8g50m50 (32432 SNPs).  The value of 0.15 of mean-r^2^ (red line) was set as a threshold for linkage equilibrium (LE) and were used to prune the 170Indvf8g50m50 dataset, remaining with 7810 independent SNPs. |
| --- |

## Population structure of invasive ALB populations in Europe

**Table S2.** F_ST_-matrix – Population differentiation on LD-pruned subset of genomic ALB SNP data with 7810 SNPs conducted in Arlequin v. 3.5.

|  | Pop-ID | 1 | 2 | 3 | 4 | 5 | 6 | 7 | 8 | 9 | 10 | 11 | 12 | 13 | 14 | 15 |
| --- | --- | --- | --- | --- | --- | --- | --- | --- | --- | --- | --- | --- | --- | --- | --- | --- |
| 1 | CH-FR-MA | 0.000 | - | + | + | + | + | + | + | + | + | + | + | + | + | + |
| 2 | CH-FRS-BR | **0.015** | 0.000 | + | + | + | + | + | + | + | + | + | + | + | + | + |
| 3 | D-BW-HLD | **0.362** | **0.372** | 0.000 | + | + | + | + | + | + | + | + | + | + | + | + |
| 4 | D-BW-WAR | **0.304** | **0.337** | 0.216 | 0.000 | + | + | + | + | + | + | + | + | + | + | - |
| 5 | D-BY-FK | **0.255** | **0.293** | 0.240 | 0.208 | 0.000 | + | + | + | + | + | + | + | + | + | + |
| 6 | D-BY-KEH | 0.227 | 0.236 | 0.134 | **0.047** | 0.148 | 0.000 | + | + | + | + | + | + | + | + | + |
| 7 | D-BY-MI | **0.377** | **0.411** | **0.255** | **0.295** | **0.311** | 0.203 | 0.000 | + | + | + | + | + | + | + | + |
| 8 | D-BY-MU | **0.271** | **0.293** | **0.274** | 0.217 | 0.229 | 0.184 | **0.268** | 0.000 | + | + | + | + | + | + | + |
| 9 | D-BY-NBB | **0.356** | **0.444** | **0.279** | **0.255** | 0.234 | 0.198 | **0.457** | **0.268** | 0.000 | + | + | + | + | + | + |
| 10 | D-BY-SB | **0.398** | **0.416** | **0.286** | 0.221 | **0.368** | 0.204 | **0.350** | **0.277** | **0.380** | 0.000 | + | + | + | + | + |
| 11 | D-ST-MB | **0.500** | **0.496** | **0.356** | **0.348** | **0.321** | **0.307** | 0.246 | **0.332** | **0.395** | **0.423** | 0.000 | + | + | + | + |
| 12 | I-BGL-TB | 0.100 | **0.093** | **0.269** | 0.165 | 0.127 | **0.090** | **0.259** | 0.198 | **0.281** | **0.287** | **0.385** | 0.000 | + | + | + |
| 13 | I-MIL-CB | **0.495** | **0.561** | **0.537** | **0.472** | **0.439** | **0.372** | **0.610** | **0.462** | **0.578** | **0.571** | **0.487** | **0.351** | 0.000 | + | + |
| 14 | I-MIL-VI | **0.487** | **0.563** | **0.497** | **0.429** | **0.369** | **0.320** | **0.575** | **0.469** | **0.544** | **0.567** | **0.422** | **0.278** | 0.149 | 0.000 | + |
| 15 | out | 0.134 | 0.141 | 0.157 | **0.056** | 0.168 | **0.036** | 0.169 | 0.126 | 0.192 | 0.119 | **0.289** | **0.073** | **0.330** | **0.313** | 0.000 |

Distance method: pairwise differences. In the lower matrix, the exact F_ST_-values are shown, while the upper matrix shows if they were significant (+) or not significant (– and red) (110 permutations); significance level = 0.05. Population labels were used as set in **Table S5**. Black boxes mark F_ST_-values >0.5, red boxes not significant F_ST_-values and bold black letters F_ST_-values >0.25, while bold green letters mark <0.1.

**Table S3.** p-values to F_ST_-values – Population differentiation on LD-pruned subset of genomic ALB SNP data with 7810 SNPs conducted in Arlequin v. 3.5.

|  | 1 | 2 | 3 | 4 | 5 | 6 | 7 | 8 | 9 | 10 | 11 | 12 | 13 | 14 | 15 |
| --- | --- | --- | --- | --- | --- | --- | --- | --- | --- | --- | --- | --- | --- | --- | --- |
| 1 | * | - | + | + | + | + | + | + | + | + | + | + | + | + | + |
| 2 | 0.12613+-0.0309 | * | + | + | + | + | + | + | + | + | + | + | + | + | + |
| 3 | 0.00000+-0.0000 | 0.00000+-0.0000 | * | + | + | + | + | + | + | + | + | + | + | + | + |
| 4 | 0.00000+-0.0000 | 0.00901+-0.0091 | 0.00901+-0.0091 | * | + | + | + | + | + | + | + | + | + | + | - |
| 5 | 0.00000+-0.0000 | 0.00000+-0.0000 | 0.00000+-0.0000 | 0.00000+-0.0000 | * | + | + | + | + | + | + | + | + | + | + |
| 6 | 0.00000+-0.0000 | 0.00000+-0.0000 | 0.00000+-0.0000 | 0.02703+-0.0194 | 0.00000+-0.0000 | * | + | + | + | + | + | + | + | + | + |
| 7 | 0.00000+-0.0000 | 0.00000+-0.0000 | 0.00000+-0.0000 | 0.00000+-0.0000 | 0.00000+-0.0000 | 0.00000+-0.0000 | * | + | + | + | + | + | + | + | + |
| 8 | 0.00000+-0.0000 | 0.00000+-0.0000 | 0.00000+-0.0000 | 0.00000+-0.0000 | 0.00000+-0.0000 | 0.00000+-0.0000 | 0.00000+-0.0000 | * | + | + | + | + | + | + | + |
| 9 | 0.00000+-0.0000 | 0.00000+-0.0000 | 0.00000+-0.0000 | 0.00000+-0.0000 | 0.00000+-0.0000 | 0.00000+-0.0000 | 0.00000+-0.0000 | 0.00000+-0.0000 | * | + | + | + | + | + | + |
| 10 | 0.00000+-0.0000 | 0.00000+-0.0000 | 0.00000+-0.0000 | 0.00000+-0.0000 | 0.00000+-0.0000 | 0.00000+-0.0000 | 0.00000+-0.0000 | 0.00000+-0.0000 | 0.00000+-0.0000 | * | + | + | + | + | + |
| 11 | 0.00000+-0.0000 | 0.00000+-0.0000 | 0.00000+-0.0000 | 0.00000+-0.0000 | 0.00000+-0.0000 | 0.00000+-0.0000 | 0.00000+-0.0000 | 0.00000+-0.0000 | 0.00000+-0.0000 | 0.00000+-0.0000 | * | + | + | + | + |
| 12 | 0.00000+-0.0000 | 0.01802+-0.0121 | 0.00000+-0.0000 | 0.00000+-0.0000 | 0.00000+-0.0000 | 0.00000+-0.0000 | 0.00000+-0.0000 | 0.00000+-0.0000 | 0.00000+-0.0000 | 0.00000+-0.0000 | 0.00000+-0.0000 | * | + | + | + |
| 13 | 0.00000+-0.0000 | 0.00000+-0.0000 | 0.00000+-0.0000 | 0.00000+-0.0000 | 0.00000+-0.0000 | 0.00000+-0.0000 | 0.00000+-0.0000 | 0.00000+-0.0000 | 0.00000+-0.0000 | 0.00000+-0.0000 | 0.00000+-0.0000 | 0.00000+-0.0000 | * | + | + |
| 14 | 0.00000+-0.0000 | 0.00901+-0.0091 | 0.00000+-0.0000 | 0.00000+-0.0000 | 0.00000+-0.0000 | 0.00000+-0.0000 | 0.00000+-0.0000 | 0.00000+-0.0000 | 0.00000+-0.0000 | 0.00000+-0.0000 | 0.00000+-0.0000 | 0.00000+-0.0000 | 0.00901+-0.0091 | * | + |
| 15 | 0.00000+-0.0000 | 0.01802+-0.0121 | 0.00000+-0.0000 | 0.10811+-0.0264 | 0.00000+-0.0000 | 0.01802+-0.0121 | 0.00000+-0.0000 | 0.00000+-0.0000 | 0.00000+-0.0000 | 0.00000+-0.0000 | 0.00000+-0.0000 | 0.01802+-0.0121 | 0.00000+-0.0000 | 0.00000+-0.0000 | * |

110 permutations, 1-15 represent the Pop-IDs shown in 2 in the same order. Population labels were used as set in **Table S5**. Values not falling below the significance level of 0.05 are marked in red.

**Table S4.** Eigenvalues from PCA analysis performed in PLINK v. 1.9 on LD-pruned 170Indvf8g50m50 subset of genomic ALB SNP-Data.

| PC | Eigenvalues | % variance explained |
| --- | --- | --- |
| 1 | 25.785 | 17.513 |
| 2 | 16.198 | 11.002 |
| 3 | 13.670 | 9.284 |
| 4 | 10.155 | 6.897 |
| 5 | 9.597 | 6.518 |
| 6 | 8.883 | 6.033 |
| 7 | 7.812 | 5.306 |
| 8 | 7.601 | 5.163 |
| 9 | 6.958 | 4.726 |
| 10 | 5.711 | 3.879 |
| 11 | 4.750 | 3.226 |
| 12 | 4.109 | 2.791 |
| 13 | 3.878 | 2.634 |
| 14 | 3.363 | 2.284 |
| 15 | 3.135 | 2.130 |
| 16 | 3.042 | 2.066 |
| 17 | 2.315 | 1.572 |
| 18 | 2.277 | 1.547 |
| 19 | 2.166 | 1.471 |
| 20 | 2.033 | 1.380 |
| 21 | 1.944 | 1.321 |
| 22 | 1.856 | 1.261 |


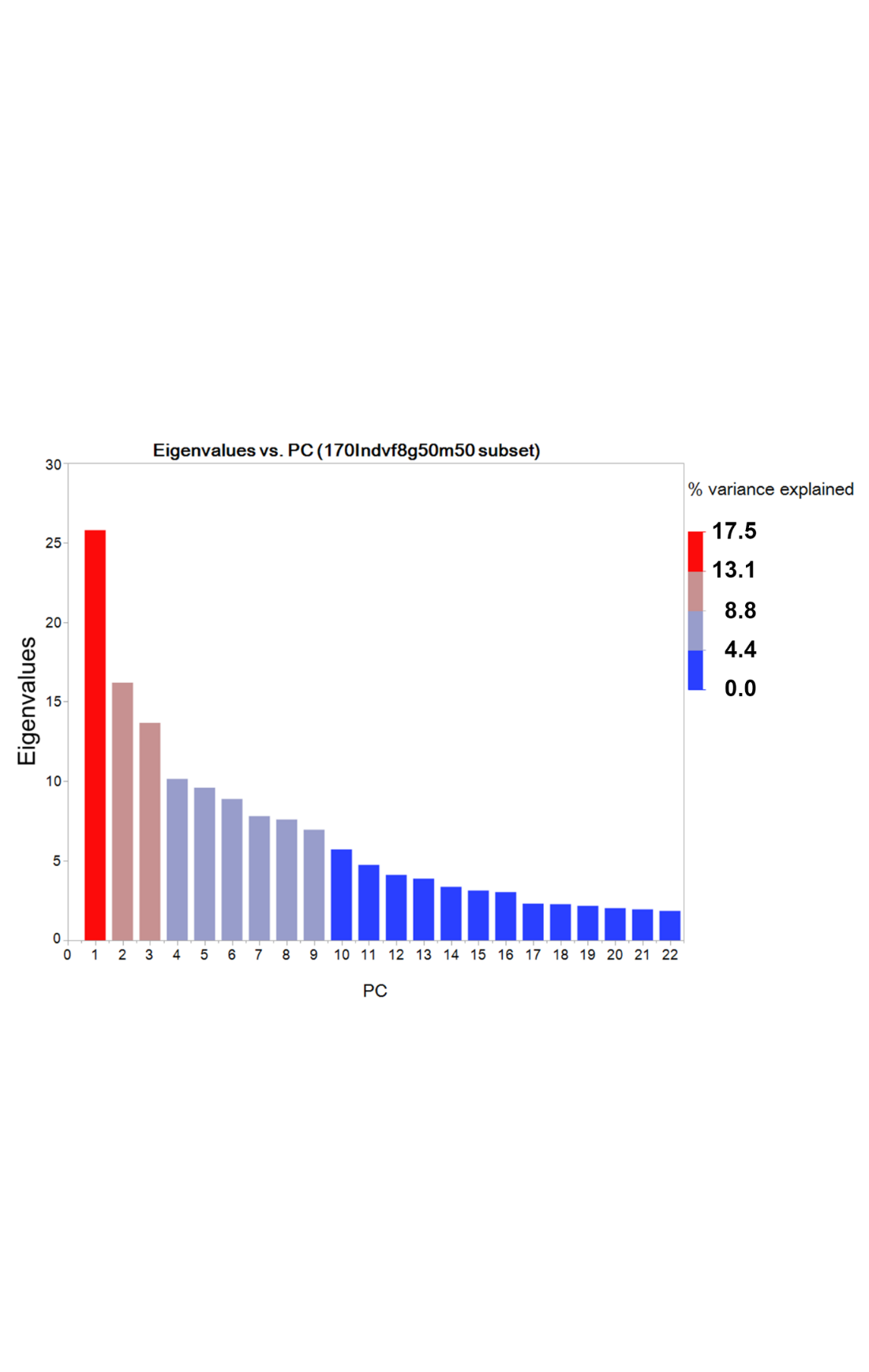


| **Figure S4.** Eigenvalues from PCA analysis performed in PLINK v. 1.9 plotted to the first 22 principal components of the LD-pruned 170Indvf8g50m50 subsets of genomic ALB SNPs. |
| --- |


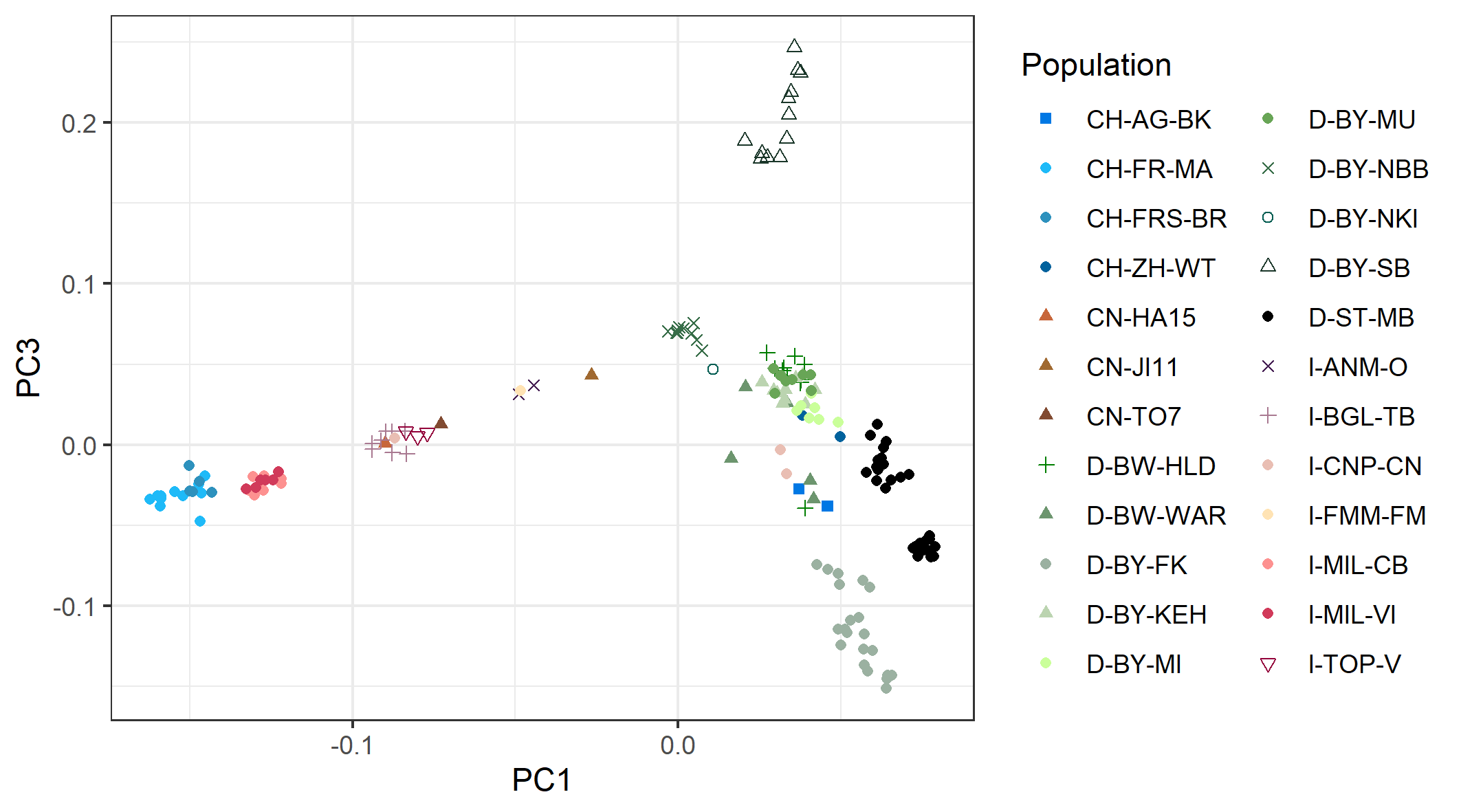


| **Figure S5.** Scatter plot of principal components PC1 (17.513%) and PC3 (9.284%) on the LD-pruned subset 170Indvf8g50m50 of genomic ALB SNP data. |
| --- |


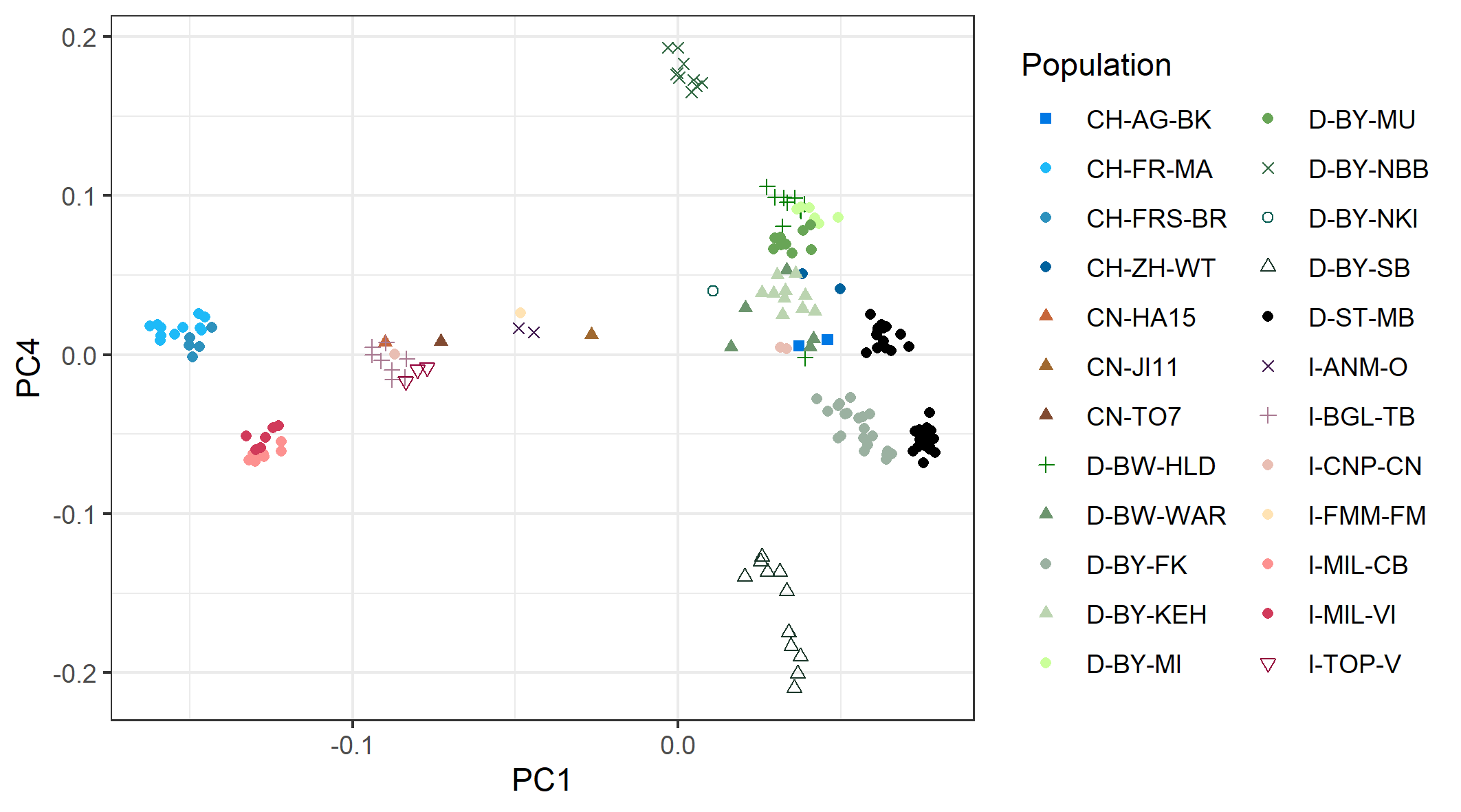


| **Figure S6.** Scatter plot of principal components PC1 (17.513%) and PC4 (6.897%) on the LD-pruned subset 170Indvf8g50m50 of genomic ALB SNP data. |
| --- |


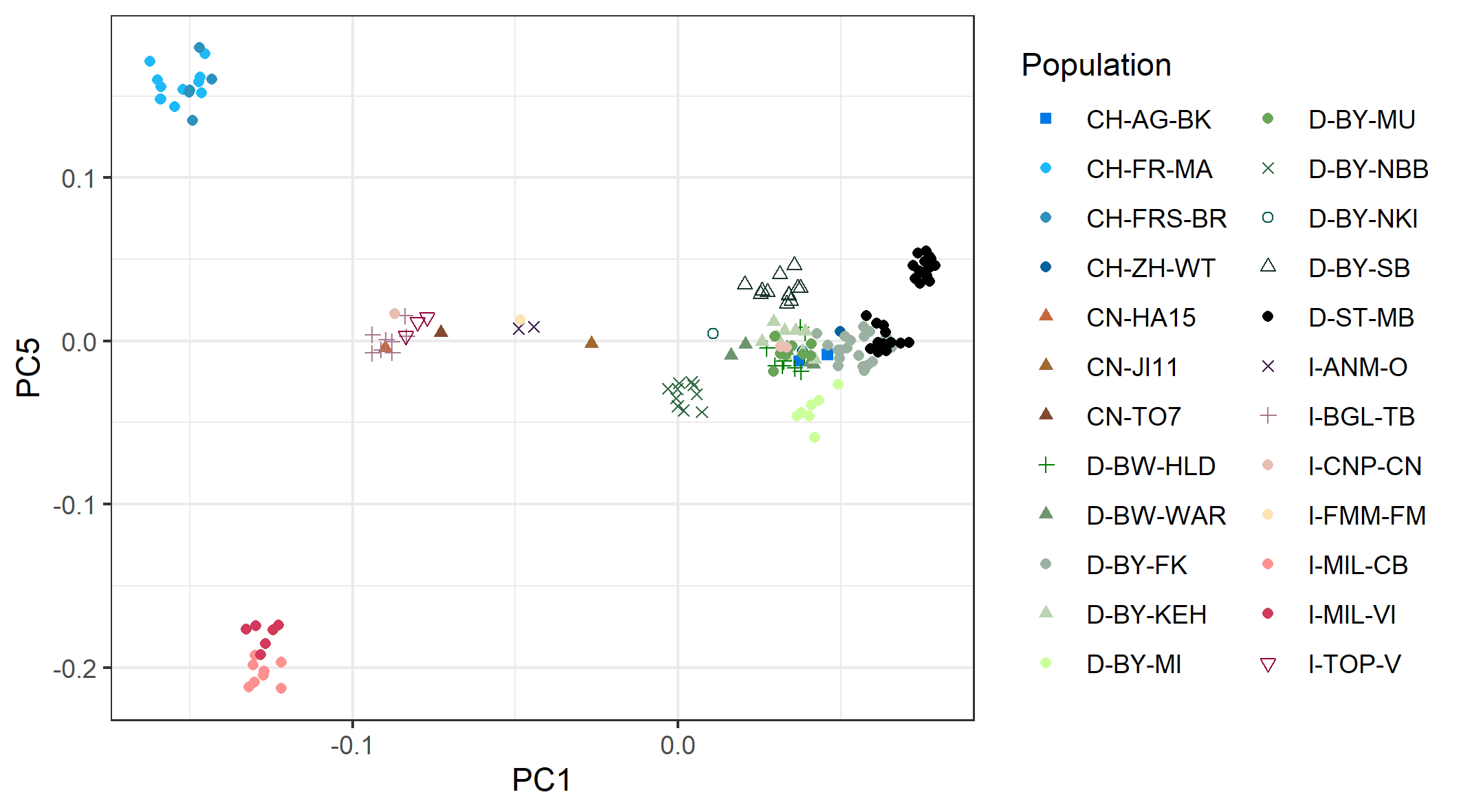


| **Figure S7.** Scatter plot of principal components PC1 (17.513%) and PC5 (6.518%) on the LD-pruned subset 170Indvf8g50m50 of genomic ALB SNP data. |
| --- |

## Ancestry of European invasive ALB populations


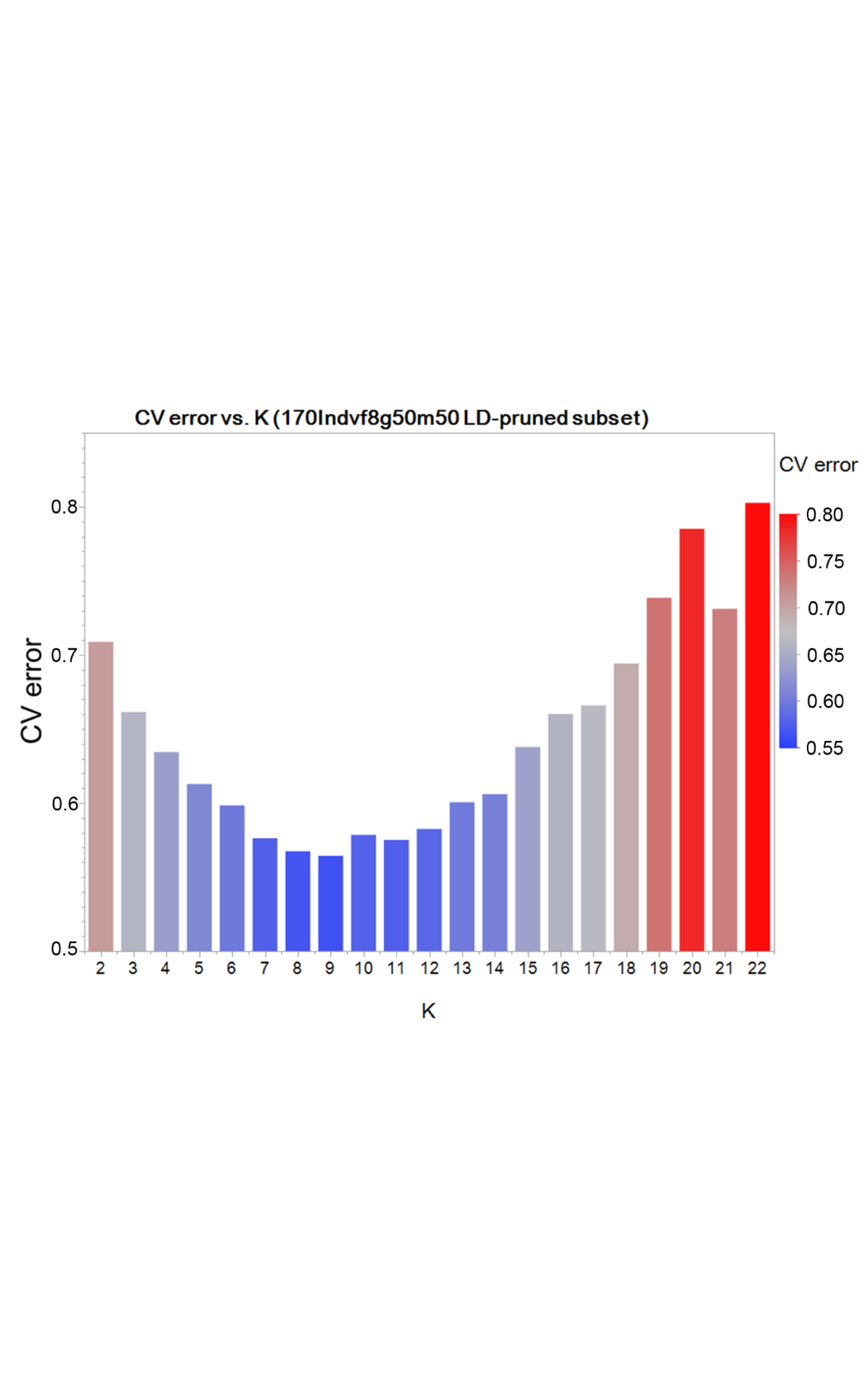


| **Figure S8.** Cross-validation errors to choose the right K-value on the ancestry in the LD-pruned 170Indvf8g50m50 ALB SNP data subset conducted in ADMIXTURE v. 1.3.0. |
| --- |


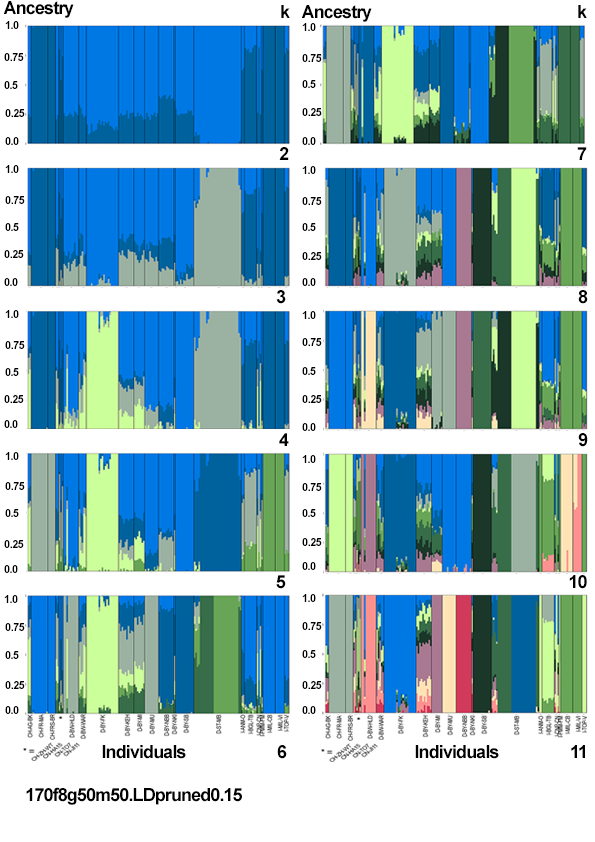


| **Figure S9.** Analysis of Ancestry of 170 ALB samples from Switzerland, China, Germany and Italy conducted in ADMIXTURE v. 1.3.0; sequential K = 2 to K = 9; 7810 SNPs.  The LD-pruned ALB SNP subset 170Indvf8g50m50 was analysed here. |
| --- |

# Methods

## Sampling of Asian long-horned beetle (ALB)

**Table S5.** ALB collection sites (alphabetically) used in this study. – (1/2)

| Country | State / province | | Location | | Lat Long | Year(s) | Pop-ID | | n | |
| --- | --- | --- | --- | --- | --- | --- | --- | --- | --- | --- |
| Switzerland (CH) | Aargau (AG) | | Berikon (BK) | | 47.349900, 8.373270 | 2015 | CH-AG-BK | | 2 | |
| Switzerland (CH) | Freiburg (FR) | | Marly (MA) | | 46.776089, 7.164610 | 2014 | CH-FR-MA | | 12 | |
| Switzerland (CH) | Freiburg Sense (FRS) | | Brünsried (BR) | | 46.759570, 7.278770 | 2013 | CH-FRS-BR | | 5 | |
| Switzerland (CH) | Zürich (ZH) | | Winterthur (WT) | | 47.499880, 8.726160 | 2012 | CH-ZH-WT | | 2 | |
| China (CN) | Heilongjiang | | Harbin (HA) | | 45.803776, 126.534966 | 2016 | CN-HA | | 1 | |
| China (CN) | Shangdong | | Jinan (JI) | | 36.651199, 117.120094 | 2016 | CN-JI | | 1 | |
| China (CN) | Inner Mongolia | | Tongliao (TO) | | 43.621910, 122.268051 | 2016 | CN-TO | | 1 | |
| Germany (D) | Baden-Württemberg (BW) | | Hildrizhausen (HLD) | | 48.625221, 8.966090 | 2016 | D-BW-HLD | | 10 | |
| Germany (D) | Baden-Württemberg (BW) | | Weil am Rhein (WAR) | | 47.592419, 7.613150 | 2015 | D-BW-WAR | | 9 | |
| Germany (D) | Bavaria (BY) | | Feldkirchen (FK) | | 48.147380, 11.730140 | 2013, 2014 | D-BY-FK | | 18 | |
| Germany (D) | Bavaria (BY) | | Feldkirchen Riem (FKR) | | 48.140630, 11.683950 | 2016 | D-BY-FK | | 4 | |
| Germany (D) | Bavaria (BY) | | Kelheim (KEH) | | 48.918610, 11.872230 | 2016 | D-BY-KEH | | 10 | |
| Germany (D) | Bavaria (BY) | | Miesbach (MI) | | 47.789420, 11.833550 | 2019 | D-BY-MI | | 7 | |
| Germany (D) | Bavaria (BY) | | Murnau (MU) | | 47.677410, 11.200950 | 2017 | D-BY-MU | | 10 | |
| Germany (D) | Bavaria (BY) | | Neubiberg (NBB) | | 48.076910, 11.657100 | 2014 | D-BY-NBB | | 10 | |
| Germany (D) | Bavaria (BY) | | Neukirchen am Inn (NKI) | | 48.519482, 13.372620 | 2012 | D-BY-NKI | | 2 | |
| Germany (D) | Bavaria (BY) | | Schönebach (SB) | | 48.308160, 10.578670 | 2015, 2018 | D-BY-SB | | 11 | |
| **Table S5**. ALB collection sites (alphabetically) used in this study. – (2/2) | | | | | | | | | | |
| Country | State / province | | Location | | Lat Long | Year(s) | Pop-ID | | n | |
| Germany (D) | Saxsony-Anhalt (ST) | | Magdeburg (MB) | | 52.131672, 11.640320 | 2015, 2018, 2019 | D-ST-MB | | 35 | |
| Italy (I) | Ancona-Marche (ANM) | Ostra (O) | | 43.614850, 13.159240 | | 2017 | | I-ANM-O | | 2 |
| Italy (I) | Bergamo-Lombardia (BGL) | Trescore-Balneario (TB) | | 45.694910, 9.846540 | | 2017, 2018 | | I-BGL-TB | | 8 |
| Italy (I) | Cuneo-Piemonte (CNP) | Cuneo (CN) | | 44.384476, 7.542671 | | 2018 | | I-CNP-CN | | 3 |
| Italy (I) | Fermo-Marche (FMM) | Fermo (FM) | | 43.158875, 13.720088 | | 2015 | | I-FMM-FM | | 2 |
| Italy (I) | Milano-Lombardia (MIL) | Corbetta (CB) | | 45.469566, 8.918932 | | 2016, 2017 | | I-MIL-CB | | 8 |
| Italy (I) | Milano-Lombardia (MIL) | Vittuone (VI) | | 45.485081, 8.958650 | | 2014, 2016 | | I-MIL-VI | | 7 |
| Italy (I) | Torino-Piemonte (TOP) | Vaie (V) | | 45.101662, 7.289800 | | 2018 | | I-TOP-V | | 3 |

n is the number of specimens per collection site (Pop-ID).
